# LAMP real-time turbidity detection for fowl adenovirus

**DOI:** 10.1186/s12917-019-2015-5

**Published:** 2019-07-23

**Authors:** Xiao-Yuan Yuan, You-Ling Wang, Kai Meng, Yu-Xia Zhang, Huai-Ying Xu, Wu Ai

**Affiliations:** Poultry Institute, Shandong Academy of Agricultural Sciences; Shandong Provincial Key Laboratory of Poultry Diseases Diagnosis and Immunology; Poultry Breeding Engineering Technology Center of Shandong Province, Ji’nan, China

**Keywords:** Adenovirus, LAMP, Real-time turbidity, Visual detection

## Abstract

**Background:**

Fowl adenovirus (FAdV) is an infectious agent that can cause jaundice, severe anaemia, dyspnoea and reproductive disorders in fowls. Since 2015, FAdV disease has been rapidly spreading among broiler chickens in China, where it has caused significant economic losses. In this study, a loop-mediated isothermal amplification (LAMP) real-time turbidity method with strong specificity to FAdV was established.

**Results:**

The established assay was specific to FAdV-4, and the lower limit of detection was 75 copies/μL of extracted DNA. The positive detection rate for the suspected tissue samples was 33.3% (14/42), which was consistent with that of the real-time PCR.

**Conclusion:**

The proposed LAMP method can quickly and accurately detect prevalent FAdV via real-time turbidity assay, thereby providing a diagnostic platform for the prevention and control of the FAdV disease.

## Background

Fowl adenovirus (FAdV) is a member of the genus *Aviadenovirus* [1]. In the last 20 years, different serotypes of FAdVs have been distributed worldwide [2–4]. In 2015, an infectious outbreak spread rapidly amongst broiler chickens in China and caused high rates of mortality. Pericardial effusions and liver lesions were the main clinical features of this disease [4, 5], and our research revealed that FAdV was the pathogen for the outbreak [6]. At present, the results of epidemiological surveys indicated that FAdV serotype 4 (FAdV-4) was the main serotype that caused this avian adenoviral epidemic.

Loop-mediated isothermal amplification (LAMP) is a simple, rapid and specific nucleic acid screening amplification method that can be performed under isothermal conditions [7]. LAMP systems enable results to be visually distinguished by the naked eye on account of the production of a large amount of magnesium pyrophosphate precipitation [8, 9]. LAMP technology has been widely used in bacterial and viral detection, drug-resistance gene detection and parasite detection [10].

In the present study, the LAMP real-time turbidity method was applied to detect FAdV-4. Subsequent clinical applications verified the applied value and practicality of the established method.

## Results

### Optimal temperature

The reaction temperature of the primers was tested in the range of 60 °C–67 °C at a temperature gradient of 1 °C. Finally, 63 °C was tested as the optimal temperature.

### Results of test specimens

**Fig. 1:**
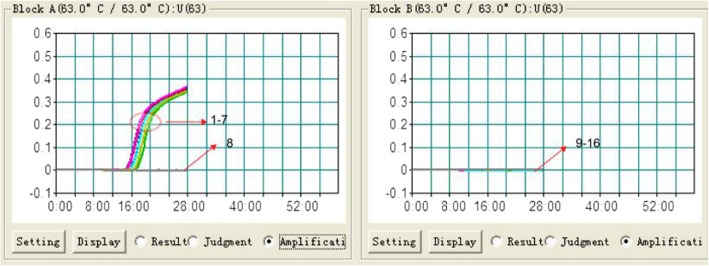
Results of test specimens (X-axis for time–minutes, Y-axis for OD 650 nm). Signals 1–7 were those of positive strains X1–X7; signal 8 was ddH_2_O; and control samples 9, 10, 11, 12, 13, 14, 15, and 16 were FAdV-8b, NDV, H9 AIV, IBV, AstV, ALV, CAV, and O78 *E. coli*, respectively

As shown in Fig. 1, LAMP based on the hexon gene can detect positive templates (signals 1–7: isolates X1–X7). Signal 8 was ddH_2_O. Control samples 9–16 were FAdV-8b serotype, Newcastle disease virus (NDV), H9 subtype Avian influenza virus (AIV), infectious bronchitis virus (IBV), astrovirus (AstV), Avian leukosis virus (ALV), chicken anaemia virus (CAV) and O78 *Escherichia coli*. No positive signal was observed for samples 8–16. The whole LAMP test was completed within 35 min.

### Limit of detection

**Fig. 2:**
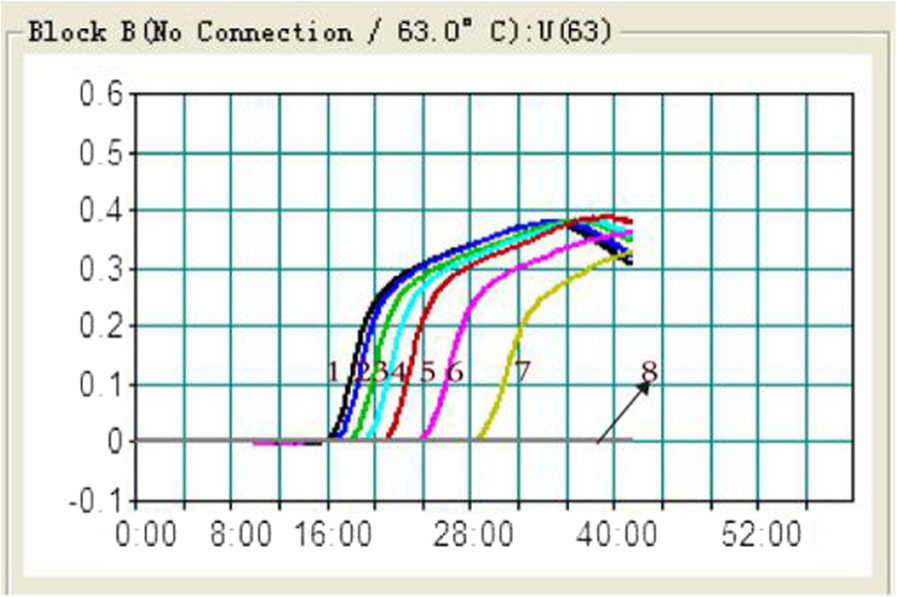
Determination of the limit of detection (X-axis for time–minutes, Y-axis for OD 650 nm). 1, the original solution with 7.5 × 10^7^ copies/μL; 2–8, dilutions of 10^1−^ 10^7^ with 7.5 × 10^6^–7.5 copies/μL. Positive amplification products were detected from Nos. 1–7. The quantification cycle (Cq) values for the real-time PCR of the templates with the same dilutions as above in LAMP (No:1–7) were 12.7, 15.8, 19.1, 22.2, 25.6, 28.8 and 31.8, respectively

**Fig. 3:**
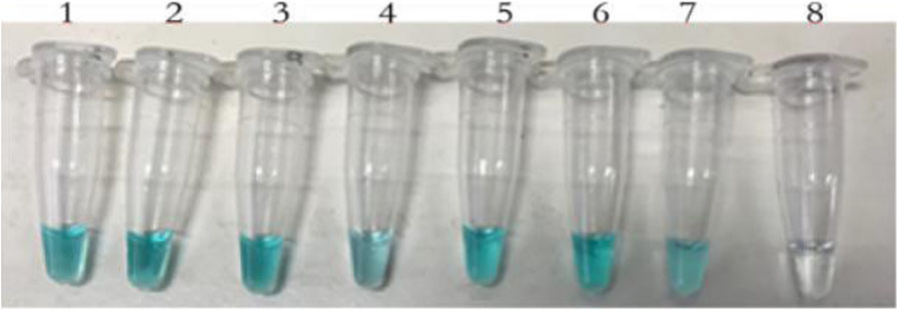
TVR dye results. 1, the original solution with 7.5 × 10^7^ copies/μL; 2–8, dilutions of 10^1^–10^7^ with 7.5 × 10^6^–7.5 copies/μL*.* From the TVR dye results shown in Fig. 3, a bright green coloration indicated positive results in 7.5 × 10^7^–7.5 × 10^1^ copies/μL, and a colorless solution (7.5 copies/μL) indicated negative results

A 10-fold dilution (total of 8 dilutions) of the DNA template was used in the LAMP reaction, and the results are shown in Figs. 2 and 3. The limit of detection of LAMP was 75 copies/μL of extracted DNA (No: 7). When the dilution template was 7.5 copies/μL of extracted DNA (No: 8), no amplification was detected. The same results were obtained by real-time PCR. A total of 20 replicates with 7.5 and 75 copies/μL DNA by LAMP and real-time PCR showed consistent results.

### Clinical application

A total of 42 samples with suspected FAdV tissues were tested using the established LAMP, and 14 positive specimens (positive detection rate, 33.3%) were detected. The results were consistent with those determined by real-time PCR (14 positives/28 negatives), and the same samples presented identical results using both assays.

## Discussion

The Avian adenovirus is a type of conditional pathogen in poultry, and it generally lacks any clinical symptoms [11, 12]. However, under certain conditions, the adenovirus can exhibit severe pathogenicity. The outbreak of the avian adenovirus disease in 2015 caused serious economic losses to agricultural production [13]. Epidemiological investigations revealed that FAdV-4 hosts typically included broiler to layer chickens, such as leghorn broilers, yellow-hair chickens and some native chickens. Infections with FAdV were often accompanied by multiple pathogens. Given the long period of traditional chicken embryo isolation, specimens with low viral content cannot be detected through conventional PCR methods. For real-time PCR, expensive instruments are needed, and reaction reagents are relatively expensive. By contrast, LAMP is a low-cost method that only requires a constant temperature state. A simple, fast and highly sensitive detection method for detecting pathogens is urgently needed [14]. The proposed method in the current work is highly sensitive and specific, and its application can be completed within only 35 min.

## Conclusion

The proposed LAMP method can quickly and sensitively detect FAdV within 35 min at a reaction temperature of 63 °C. Therefore, this method was deemed clinically convenient for clinical application.

## Methods

### Strains

Seven FAdV-4 (X1–X7) strains isolated from 2015 to 2017 in China were used for LAMP. The control strains (Nos. 9–16) included FAdV-8b serotype, NDV, H9 subtype AIV, IBV, AstV, ALV, CAV and O78 *E. coli*. These strains were isolated and identified by Poultry Institute, Shandong, China. All tissue samples in this study were derived from commercial animal farms in China.

### Design of primers

The sequences of FAdV-4 epidemic strains were screened for conservative regions. The LAMP primers were an exact match to 99.9% of the FAdV-4 sequences in GenBank (*n* = 102). Specific LAMP primers were designed on the basis of the conserved regions of hexon genes (GenBank No: KX421404) of FAdV-4 (software website: http://primerexplorer.jp/e/). The sequence to be amplified was divided into six regions to design four primers (the inner primers FIP and BIP and the outer primers F3 and B3). Two loop primers (LB and LF) were designed on the basis of two stem-loop regions. The primers were synthesized and purified by Aoike Biology Co., China. The primer sequences are shown in Table 1.

**Table 1:** LAMP primer sets for FAdV

Primer	Sequence (5′–3′)	Position
F3	AGTCTGGGCAACGACCTG	1681–1698
B3	GAATGTTGATGGTGAGGGC	1897–1879
FIP	TTACTGGTGTTGTGATCCATGGGC-GCCAGCATCATCTACAACGAG	1778–1755 1711–1731
BIP	CTGATGCTGAGAAACGCCACC-GGGCACCGAGTATAGAGC	1789–1809 1866–1849
LF	AAGTTGGCCATGAGGTTCA	1751–1733
LB	GATCAGACCTTCGTGGACT	1813–1831

### LAMP reaction

The DNAs or RNAs of FAdVs (1–7) and the control strains (9–16) were extracted according to the instructions of the DNA/RNA extraction kit (BioTek Co., China). RNA specimens were reverse-transcribed to cDNA using the RT Mix Kit (Takara, China).

The LAMP kit was obtained from Eiken Chemical Co., Japan. The LAMP reaction system (25 μL) contained 12.5 μL of 2 × buffer, 1 μL of enzyme mixture, FIP and BIP (40 pmol each), F3 and B3 (10 pmol each), LB and LF (20 pmol each) and 2 μL of the template. The mixture was placed at 63 °Cfor 60 min, and ddH_2_O was used as the control. Three replicates of each sample were run for LAMP and real-time PCR.

Real-time turbidimetry and Tris-EDTA visual reagent (TVR, patent-pending, JXD Co., China) were used to evaluate the reaction results. White precipitates of Mg_2_P_2_O_7_ were produced during the LAMP reaction, and thus, the turbidity of the reaction tube was monitored every 6 s by a real-time turbidimeter (La-320, Eiken Chemical Co., Japan). A curve was then drawn to judge whether the reaction was positive. The addition of the TVR indicator to the reaction system resulted in different colors. Once double-chain nucleic acids appear in the amplification system, the dye groups can bind to them and show a bright green color, indicating a positive result; a colorless solution is deemed a negative result.

### Determination for the limit of detection

The 10-fold dilution of DNAs for strain X1, with initial DNA concentration of 7.5 × 10^7^ copies/μL (NanoDrop ND-1000, Nanodrop Co., USA), and eight dilution gradients were used to verify the limit of detection of LAMP. Twenty replicates with 7.5 and 75 copies/μL of DNA were run for the limit of detection of LAMP and real-time PCR to confirm reproducibility.

The above dilutions were tested with real-time PCR as a contrast test method according to the following procedures. Primer Express 3.0 software was used to design the real-time PCR primers and probe for the conserved sequence of prevalent FAdV-4 strains. Hairpins, self-dimers and cross-dimers in primer pairs were avoided. The forward primer was 5′-CTAGGGTTCTGAACTTTG-3′, the reverse primer was 5′-CGGTAAACATTTCAAGGA-3′, and the TaqMan probe was 5′-FAM-TGACGCCAGTTTCGCTTTCG-Eclipse-3′. Real-time PCR was conducted in a CFX-96 system from BioRad Inc., USA. An optimal real-time PCR system of 25 μL included the following: 12.5 μL of 2 × Premix Ex (for qPCR), 1 μL of each primer (10 μM), 2 μL of the template. The optimal reaction was as follows: 95 °C for 2 min, 40 cycles of 94°Cfor 5 s and 55°Cfor 30 s. The fluorescence signal was collected at 60 °C. The real-time PCR assay was specific to FAdV-4 and not to FAdV-8b serotype, NDV, H9 subtype AIV, IBV, AstV, ALV and CAV.

### Detection of clinical samples

A total of 42 tissue samples (22 liver samples, 12 kidney samples and 8 lung samples from different individuals) with suspected FAdV were tested using the established LAMP method. Viral DNA was extracted by the DNA extraction kit (AU52111) from BioTek Co., China. Real-time PCR was also used as a contrast detection method.

## Data Availability

The datasets analyzed in the current study are available from the corresponding author upon reasonable request.

## Abbreviations

AIV: Avian influenza virus
ALV: Avian leukosis virus
AstV: Astrovirus
CAV: Chicken anaemia Virus
Cq: Quantification cycle
*E. coli*: *Escherichia coli*
FAdV: Fowl adenovirus
IBV: Infectious bronchitis virus
LAMP: Loop-mediated isothermal amplification
NDV: Newcastle disease virus
PCR: Polymerase chain reaction
TVR: Tris-EDTA visual reagent
